# Neuroprotective Potential of Tamarillo (*Cyphomandra betacea*) Epicarp Extracts Obtained by Sustainable Extraction Process

**DOI:** 10.3389/fnut.2021.769617

**Published:** 2021-11-15

**Authors:** Zully Jimena Suárez-Montenegro, Diego Ballesteros-Vivas, Rocío Gallego, Alberto Valdés, Jose David Sánchez-Martínez, Fabián Parada-Alfonso, Elena Ibáñez, Alejandro Cifuentes

**Affiliations:** ^1^Foodomics Laboratory, Institute of Food Science Research (CIAL, CSIC), Madrid, Spain; ^2^Departamento de Procesos Industriales, Facultad de Ingenieria Agroindustrial, Universidad de Nariño, Pasto, Colombia; ^3^High Pressure Laboratory, Departamento de Química, Facultad de Ciencias, Food Chemistry Research Group, Universidad Nacional de Colombia, Bogotá, Colombia; ^4^Departamento de Nutrición y Bioquímica, Facultad de Ciencias, Pontificia Universidad Javeriana, Bogotá, Colombia

**Keywords:** neuroprotective activity, Alzheimer's disease, *Cyphomandra betacea*, tropical fruit by-product, green extraction, pressurized liquid extraction, polyphenol extracts

## Abstract

Tamarillo (*Cyphomandra betacea* (Cav.) Sendt.), or tree tomato, is a tropical fruit from the Andean region of South America; it is highly rich in vitamins, minerals, and polyphenolic compounds. In this study, extracts from tamarillo epicarp (TE) were obtained by pressurized liquid extraction (PLE), and their *in-vitro* neuroprotective potential was assessed. A central composite design with response surface methodology was performed to optimize PLE as a function of solvent composition and temperature. Selected response variables were extraction yield, total phenolic content (TPC), total flavonoid content (TFC), total carotenoid content (TCC), antioxidant (ABTS), and anti-inflammatory (LOX) activities, and anti-acetylcholinesterase (AChE) inhibitory capacity. According to the desirability function, the optimal conditions were 100% ethanol and 180°C with a 0.87 desirability value. Next, the anti-butyrylcholinesterase enzyme (BChE), reactive oxygen species (ROS), and reactive nitrogen species (RNS) inhibition as well as cytotoxicity in HK-2, THP-1 monocytes, and SH-5YSY neuroblastoma cell lines were studied for the TE extract obtained under optimized conditions. The optimum TE extract provided the following results: extraction yield (36.25%), TPC (92.09 mg GAE/g extract), TFC (4.4 mg QE/g extract), TCC (107.15 mg CE/g extract), antioxidant capacity (ABTS, IC_50_ = 6.33 mg/ml extract), LOX (IC_50_ = 48.3 mg/ml extract), and AChE (IC_50_ = 97.46 mg/ml extract), and showed no toxicity at concentration up to 120 μg/ml extract for all the tested cell lines. Finally, chemical characterization by liquid chromatography-tandem mass spectrometry (UHPLC-q-TOF-MS/MS) of the optimum TE extract exhibited an important presence of hydroxycinnamic acid derivatives and other phenolic acids as well as quercetin hexoside and rutin, as main metabolites responsible for the observed biological properties. All these results suggested that TE, which represents between 8 and 15% of the total fruit, could become a promising natural by-product with a potential “multitarget” activity against Alzheimer's disease.

## Introduction

Tamarillo (*Cyphomandra betacea (Cav.) Sendt*.), also known as the tree tomato, is a solanaceous fruit growing in subtropical climates of South America, especially in countries that are crossed by the Andes Mountain, such as Bolivia, Chile, Colombia, Ecuador, Perú, and Venezuela, and is being considered as an Andean crop whose commercial production occurs in Colombia, Perú, and Ecuador (1). Tamarillo fruit is a promising bioresource since it is the fourth tropical fruit produced in Colombia, after pineapple, mango, and avocado (2). Because it grows in a wide range of altitudes (from 1,500 to 3,000 m) and easily adapts in frost-free climatic areas, this emerging crop has been introduced in agricultural economies of many countries, such as Argentina, Brazil, New Zealand, Australia, South-east Asia, Italy, Jamaica, Haiti, Mexico, Puerto Rico, Costa Rica, East Africa, Spain, and Portugal (1, 3).

**Figure 1:**
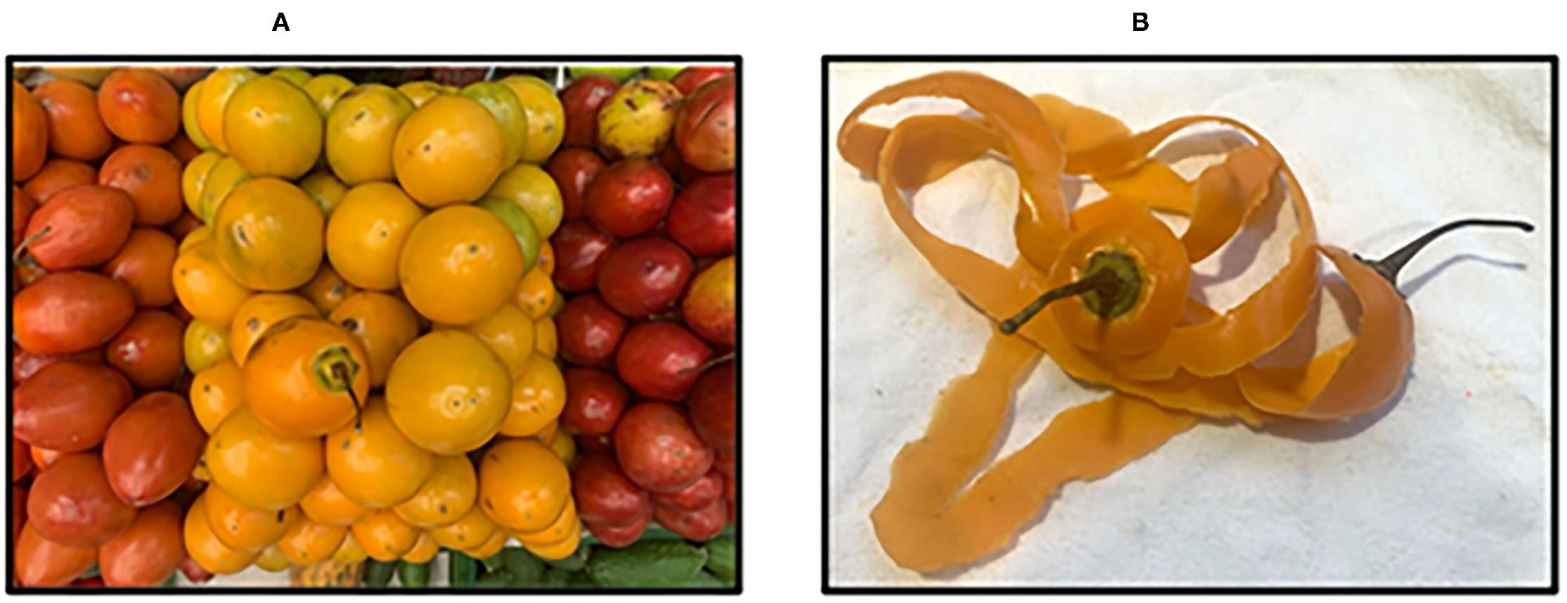
**(A)** Tamarillo fruit ecotypes (red, yellow, and purple) and **(B)** tamarillo fruit epicarp (yellow ecotype).

Tamarillo fruit is an elliptical berry with an ovoid shape and between 5–10 cm long and 5–8 cm in diameter. The fruit is covered by a thick and bitter epicarp with purple, red, or yellow tones according to ecotype (4) and represents around 8–15% of waste biomass within industrial processing (5, 6) (see Figures 1A,B). The fruit is rich in minerals such as potassium, calcium, magnesium, iron, zinc, among others; non-starch polysaccharides, dietary fiber, vitamins A, C, B_6_, E, and K, carotenoids, and polyphenols (3, 4, 7). Recently, Martin et al. conducted a full study by Raman spectroscopy and Fourier transforms infrared (FTIR) in attenuated total reflectance configuration (FTIR-ATR) in order to identify the type and relative amount of metabolites present in tamarillo pulp, epicarp, and seeds as a guide to elucidate its nutraceutical potential (8).

Several studies have reported the agronomical, physical, chemical, and nutritional characteristics of tamarillo (3, 7, 9, 10). Some biological activities have been described for the tamarillo pulp, mainly focused on treating obesity (11), or having antimicrobial (12), anti-inflammatory (11), antioxidant (10, 13–16), and antitumoral (15) properties. Moreover, only one study was focused on the nutritional properties of tamarillo seeds (17), while a few studies can be found related to the activity of tamarillo epicarp (TE) against lipid oxidation (18) and antibacterial potential (19).

At present, data of lifespan (especially in developed countries) have increased, which brings with it a great challenge: healthy aging. However, with elder age, the neurodegenerative process also advances as a natural consequence of brain aging. Manifestations, such as loss of memory, cognitive skills, and language, are exacerbated by the presence of hallmarks, such as oxidative damage, neuroinflammation, and accumulation of toxic amyloid peptides. Among the different neurodegenerative diseases (NDs), Alzheimer's disease (AD) is one of the most frequent; its development is directly related to the presence of extracellular plaques composed of amyloid-beta (Aβ) protein, intraneuronal tau (τ) in its hyperphosphorylated form, significant loss of synaptic connectivity and neuronal death, promoting brain atrophy. Chemical-based therapies have been used to alleviate some symptoms, although no treatment for AD is available yet; as a result, there is an increasing interest in developing new supplements based on natural extracts that can help in slowing down the neurodegeneration process. In this sense, it is worth mentioning the interest in nutraceutical compounds for a “multitarget” therapy of AD, which has been recently recognized as one important possibility for the future control of this disease (20). Some food-related sources and components have been already highlighted as potential anti-AD agents both *in vitro* and *in vivo* (21), namely, polyphenols and alkaloids (22), carbohydrates (23), and proteins (24) contained in plants and marine organisms (25). Other interesting sources of bioactive compounds with promising biological activities are food by-products that can be employed as a cheap source of high-added value compounds possessing pharmacological activity against neurodegenerative disorders.

To accomplish the recovery of bioactive compounds from food waste and its application in the food, pharmaceutical, or cosmetic fields, sustainable processes are preferred according to the United Nations Sustainable Development Goals (SDGs). Therefore, eco-friendly processes are intended to comply with the following characteristics: (1) preserving the biological activity of metabolites; (2) being technically efficient (high selectivity and low consumption of energy and solvents); (3) being innocuous (no hazard for the consumer). Among them, pressurized liquid extraction (PLE) is a useful technology to obtain bioactive metabolites from different solid matrices. This technique works at high pressure and temperature, and it can be employed with a wide range of green solvents promoting an important reduction in extraction time and energy consumption, as well as high selectivity, being a suitable green technique for natural sources and by-products.

In summary, the aim of this study was to evaluate the neuroprotective potential of different extracts from TE obtained by PLE and to optimize the operating conditions to attain an extract with potential “multitarget” activity against AD (antioxidant, anti-inflammatory, and anti-cholinergic). In addition, cytotoxicity in HK-2, THP-1 monocytes, and SH-5YSY neuroblastoma cell lines was assessed for the most promising TE extract.

## Materials and Methods

### Sample Preparation

Tamarillo (*Cyphomandra betacea*, yellow ecotype) fruit (3 kg) was acquired in a local market in Colombia. The TE was removed manually (600 g) and washed with distilled water. Then, it was dried at room temperature for 72 h, grounded, and sieved through 50 mesh (average particle size: 0.278 ± 0.008 mm); finally, 90 g of dry TE was obtained.

### Reagents

Trizma hydrochloride (Tris-HCl), bovine serum albumin (BSA), potassium phosphate monobasic (KH_2_PO_4_) ≥ 99%, sodium phosphate dibasic (NaH_2_PO_4_) ≥ 99%, potassium persulfate (K_2_S_2_O_8_) ≥ 99%, sodium carbonate (Na_2_CO_3_) ≥ 99%, sodium nitroprusside dehydrate (SNP), fluorescein sodium salt, sulfanilamide, naphthylethylene diamine dihydrochloride, phosphoric acid, gallic acid, quercetin, linoleic acid (LA), and formic acid 99% were purchased from VWR Chemicals (EC); 7-fluorobenzofurazan-4-sulfonamide (ABD-F) 98% was acquired from Alfa Aesar (Kandel, Germany). Acetylcholinesterase (AChE) from *Electrophorus electricus* (electric eel) type VI-S, butyrylcholinesterase (BChE) from equine serum, 2,2-azino-bis (3-ethylbenzothiazoline-6-sulphonic acid) (ABTS^·+^), 3-(4,5-dimethylthiazol-2-yl)-2,5-diphenyltetrazolium bromide (MTT), phorbol 12-myristate 13-acetate (PMA), and β-mercaptoethanol were purchased from Sigma-Aldrich (Madrid, Spain). Acetylthiocholine iodide (ATCI) ≥ 99%, butyrylthiocoline iodide (BTCI) ≥ 99%, lipoxidase from glycine max (soybean), type 1-B and (±)-6-hydroxy-2,5,7,8-tetramethylchromane-2-carboxylic acid (Trolox) > 97%, were obtained from Sigma-Aldrich (Madrid, Spain). Folin-Ciocalteu's phenol reagent and aluminum chloride hexahydrate were purchased from Merck (Darmstadt, Germany). Galantamine hydrobromide, purity > 98%, was purchased from TCI Chemicals (Tokyo, Japan). HPLC-grade such as methanol, ethanol, and acetonitrile were purchased from VWR Chemicals (Barcelona, Spain), and ethyl acetate was purchased from Scharlau (Barcelona, Spain). Dichloromethane was purchased from Fluka AG (Buchs, Switzerland). MS grade acetonitrile and water from LabScan (Dublin, Ireland) were employed for UHPLC-q-TOF-MS. Ultrapure water was obtained from a Millipore system (Billerica, MA, United States). All the 96-well microplate assays were performed in a spectrophotometer and fluorescent reader (Synergy HT, BioTek Instruments, Winooski, VT, United States).

### Pressurized Liquid Extraction (PLE)

Tamarillo epicarp extracts were obtained by a PLE process using a Dionex accelerated solvent extractor (ASE 200; Sunnyvale, CA, United States) at 1,500 ± 200 psi and 20 min as extraction time, as described in our previous study (26). Extraction conditions (temperature and solvent composition) were optimized according to a central composite design (CCD) and as described in the next section. Mixtures of water:ethanol, including a fixed percentage of formic acid (2%), were selected as previously suggested (27).

Near 200 mg d.w. of TE (for experiments T11-T13, when no EtOH was employed) and near 1,000 mg d.w. of TE (for the rest of experiments, including EtOH in the solvent composition) were subjected to PLE under different conditions obtaining nine extracts (T11-T13, T21-T23, T31-T33); five replicates of extract T22 were made to evaluate yield reproducibility. Under selected extraction conditions, two phases were obtained, a soluble phase and an insoluble phase (pectin), except when EtOH-formic acid 98:2 was used at 60°C (extract T31); under these conditions, only one phase was found (pectin was not extracted). Precipitates were separated by centrifugation (Eppendorf centrifuge 5804R; Eppendorf, Hamburg, Germany) at 10,000 rpm, 10°C, 30 min, and the supernatant was saved (S1); the remaining solid was washed with EtOH (5 ml) and centrifugated, obtaining a second supernatant (S2) that was mixed with S1, constituting each of the obtained extracts (T11 to T33). Samples extracted were first dried under N_2_ and then freeze-dried in a freeze-dryer (Lyobeta; Telstar, Terrassa, Spain).

Total yield was calculated as the ratio between the mass of extract at dry basis (x) and the mass of the dry sample was fed into the extraction cell (y), according to Equation 1:


(1) $$\begin{array}{l} \text{Extraction yield }\%\;(\text{w}/\text{dw})=\text{x}(\text{extract mass})/\text{y}(\text{initial mass})\\ \;\times 100 \end{array}$$

### Response Surface Methodology

The optimization of the extraction of bioactive compounds from TE by PLE was carried out using a CCD with response surface methodology (RSM). Two factors were considered at three levels: solvent composition (percentage of water in the mixture H_2_O/EtOH: 0, 50, and 100% v/v; considering a fixed 2% of formic acid) and temperature (60, 120, and 180°C). Selected response variables were extraction yield, total phenolic content (TPC), total flavonoid content (TFC), total carotenoid content (TCC), antioxidant (ABTS) and anti-inflammatory (LOX) activities, and anti-cholinergic (AChE) inhibitory capacity. The experimental data were fitted to a quadratic model for each response variable, Y_i_:


(2) $$\begin{array}{l} Y_{i}=\beta_{0}+\beta_{1}SC+\beta_{2}T+\beta_{1,2}T\times SC+\beta_{1,1}SC^{2}\\ \;+\;\beta_{2,2}T^{2}+error \end{array}$$

where *SC* is the solvent composition, *T* is the temperature, β_0_ is the intercept, β_1_, and β_2_ are the linear coefficients, β_1,2_ is the linear-by-linear interaction coefficient, β_1,1_ and β_2,2_ are the quadratic coefficients, and *error* is the error variable. STATISTICA 12 (Stat Soft, Inc., Tulsa, OK 74104, United States) was used for experimental design, data analysis, and multiple response optimization. The adequacy of the models was determined by the coefficient of regression (R^2^) and the *F*-test value (*F*-value) obtained from the analysis of variance (ANOVA). In addition, multiple response optimization was performed by the combination of experimental factors, looking to maximize the desirability function.

### *In vitro* Assays

#### Total Phenolic Content (TPC)

Total phenolic content determination was performed by Folin–Ciocalteu method according to García Martinez et al. (28), with some modifications. A calibration curve was constructed using gallic acid (0–1,000 μg gallic acid per ml EtOH) as a chemical standard. An aliquot of 10 μl of TE extracts solution at a concentration range of 5–12 mg/ml in EtOH was mixed with 600 μl of H_2_O and 50 μl of Folin–Ciocalteu. After 1 min of agitation, 150 μl of Na_2_CO_3_ (20% w/v) was added, and the volume was completed to 1 ml with 190 μl of H_2_O. After 120 min of incubation at room temperature, 300 μl of each mixture was placed in a 96-well microplate spectrophotometer reader and the absorbance at 760 nm was measured. TPCs were expressed as gallic acid equivalents per gram extract (mg GAE/g of extract). All measurements were done in triplicate.

#### Total Flavonoid Content (TFC)

Total flavonoid determination was performed according to Kaushal et al. (29). A calibration curve from 0 to 14 μg/ml of a standard solution of quercetin (24 μg/ml) was constructed in order to measure the total flavonoids (or quercetin), expressed in milligram quercetin per gram extract (mg QE/g extract); 100 μl of TE extracts (with a concentration range of 5 to 12 mg extract/ml EtOH), 140 μl of MeOH, and 60 μl of anhydrous aluminum chloride (8 mM) were placed in each well and the absorbance was measured by spectrophotometry using a 96-well plate reader after 30 min at a wavelength 425 nm. All the measurements were done in triplicate.

#### Total Carotenoid Content (TCC)

Total carotenoid determination was performed according to Gilbert-López et al. (30). For this purpose, a standard solution of β-carotene as a reference carotenoid present in tamarillo was used as a chemical standard. A calibration curve at a concentration range of 0.172–22 μg/ml with R^2^ = 0.9935 was constructed in order to calculate the TTC expressed as milligram carotenoids per gram extract (mg CE/g extract). The TE extracts were dissolved in EtOH at a concentration range from 5 to 12 mg extract/ml EtOH, and then 300 μl was placed in each well and the absorbance was recorded by spectrophotometry using a 96-well plate reader at a wavelength of 470 nm. All the measurements were performed in triplicate.

#### *In vitro* Biological Activities Associated With AD

A total of nine TE extracts were tested using a battery of *in vitro* assays associated with neurodegenerative processes, such as antioxidant (ABTS^·+^) scavenging capacity, anti-inflammatory activity (LOX), and inhibition of acetylcholinesterase enzyme (AChE). For the most promising extract (T33), scavenging capacity against ROS and RNS species was measured by oxygen radical absorbance capacity (ORAC) and nitric oxide (NO^·^) radical, respectively. Moreover, butyrylcholinesterase enzyme (BChE) inhibition capacity and cell toxicity using the MTT assay were also performed for the T33 extract. All the assays were developed with a methodology previously described by Suárez Montenegro et al. (31).

##### Antioxidant Activity

- ABTS Scavenging Activity

ABTS^·+^ radical scavenging assay was performed according to Re et al. (32), with modifications. TE at six different concentrations of each sample [from 2.9 to 29 μg/ml EtOH/H_2_O (1:1, v/v)] was used, giving a linear response between 20 and 80% of the control absorbance. All the measurements were performed in triplicate. Equation (3) represents the percentage of ABTS^·+^ inhibition due to TE extracts and Trolox compared with the ABTS control.


(3) $$\%Inh=\frac{A_{ABTS\;control}-\left(A_{sample}-A_{sample\;blank}\right)}{A_{ABTS\;control}}\;x\;100$$

where A _ABTScontrol_ is absorbance of radical at t = 0 min; A_sample_, represents the mean of absorbance readings of TE samples, and A_Sampleblank_ is the absorbance of the sample without ABTS. The capacity of free radical scavenging was expressed by IC_50_ value, and the results represent the mean inhibitory concentration (in μg/ml) of TE against ABTS^·+^ radical.

- ROS Scavenging Capacity

Reactive oxygen species scavenging capacity was carried out by means of the ORAC method, according to Ou et al. (33) for the T33 sample. In this case, a concentration range of extract from 0.62 to 6.2 μg/ml in EtOH/H_2_O (1:9, v/v) was measured. The capacity of the T33 extract to scavenge peroxyl radicals generated by spontaneous decomposition of 2,20-azobis (2-amidinopropane) dihydrochloride (AAPH) was calculated through the inhibition percentage of the difference between the area under the curve (AUC) of fluorescence decay in the presence (AUC_sample_) or absence (AUC_control_) of the sample by Equation (4). The measurements were carried out in triplicate. Trolox and rosemary extracts were used as chemical and natural positive controls, respectively.


(4) $$\%Inh=\frac{AUC_{Control}-AUC_{sample}}{AUC_{control}}\;x\;100$$

AUC was calculated by means of Equation (5).


(5) $$AUC=0.5+\sum \frac{f_{i}}{f_{o}}$$

where *f*_0_ and *f*_*i*_ are fluorescence at *t* = 0 and every 5 min, respectively.

- RNS Scavenging Capacity

Reactive nitrogen species scavenging capacity was measured following the NO^·^ radical scavenging assay according to Ho et al. (34) and based on the Griess reaction. A concentration range from 70 to 700 μg/ml of the T33 extract in EtOH/H_2_O (1:3, v/v) was tested. Each measurement was carried out in triplicate, and ascorbic acid and rosemary extract were used as chemical and natural positive controls, respectively. The NO scavenging capacity of the extract was expressed through inhibition % calculated as described in Equation (3).

##### Anti-inflammatory Activity

Anti-inflammatory activity was measured by lipoxidase (LOX) inhibitory capacity enzyme by means of the fluorescence-based enzyme kinetic method, using fluorescein as a probe according to Whent et al. (35). In this case, TE at seven different concentrations of each sample (from 21.4 to 214 μg/ml) in EtOH/H_2_O (0.25:1) was measured. Equation (6) represents the percentage of inhibition of the sample compared with the negative control, where V_1_ and V_0_ are mean velocities obtained for LOX in the presence and absence of inhibitors. Quercetin and rosemary extract were used as chemical and natural positive controls, respectively. Each measurement was carried out in triplicate, and the results are expressed as mean ± standard deviation. IC_50_ value represents the concentration of quercetin or TE extract that produced 50% of enzyme inhibition capacity as compared with control (without inhibitors).


(6) $$\%\;Inh=\;\frac{V_{0}-\;V_{1}}{V_{0}}\;x\;100$$

##### Anti-cholinergic Activity: AChE and BChE

The anti-cholinergic activity was evaluated by means of AChE and BChE inhibitory capacity enzymes of TE extracts, based on Ellman's method (1961) (36), modified by fluorescent enzyme kinetics study using ABD-F as a fluorescent probe (37). Seven different concentrations (17–167 μg/ml for AChE, and 37 to 370 for BChE) in EtOH/H_2_O (1:1, v/v) were measured. Equation (6) represents the percentage of inhibition of sample compared with negative control, where V_1_ and V_0_ are mean velocities obtained for AChE (or BChE) in the presence and absence of inhibitors. Each measurement was carried out in triplicate, and the results are expressed as mean ± standard deviation. IC_50_ value represents the concentration (in μg/ml) of galantamine or TE extract that produced 50% of cholinergic enzyme inhibition capacity as compared with control (without inhibitors), so it means lower IC_50_ concentrations exhibit a major inhibitory potency compared with higher IC_50_ values.

### Cell Culture Conditions and Toxicity Assays

The *in vitro* toxicity evaluation of the selected TE extract (T33) was tested on three different cell lines: human proximal tubular epithelial cells (HK-2), human THP-1 monocytes, and human SH-5YSY neuroblastoma cells (all purchased from ATCC^®^, Rockville, MD, USA). Cell culture medium Dulbecco's Modified Eagle Medium Nutrient Mixture (DMEM/Ham's F12) and Roswell Park Memorial Institute (RPMI 1640) were acquired from Thermo Fisher Scientific (Grand Island, NY, United States). Also, fetal bovine serum (FBS), L-glutamine, penicillin, streptomycin, antibiotic-antimycotic solution, insulin-transferrine-selenium (ITS), and β-mercaptoethanol were purchased from Thermo Fisher Scientific (Grand Island, NY, United States). Phorbol 12-myristate 13-acetate (PMA) was acquired from Sigma-Aldrich (Madrid, Spain). Retinoic acid (RA) was purchased from Glentham Life Science (Corsham, United Kindgdom), whereas brain-derived neurotrophic factor (BDNF) was acquired from Bachem AG (Bubendorf, Switzerland).

HK-2 cells were grown according to the methodology previously reported in Suárez Montenegro et al. (31). For this purpose, cells were plated in 96-well plates at a density of 5 × 10^3^ cells/well and, after 24 h, for proper attachment, different concentrations of the tamarillo extract (7.5 to 120 μg/ml) were added to the cells, which were then incubated for 24 h. The viability of the cells was then determined by MTT assay (38). Briefly, after 24 h of exposition to the T33 extract, the culture medium was removed, and the cells were incubated with 0.5 mg/ml MTT for 3 h at 37°C. Dimethyl sulfoxide (DMSO) was added to solubilize formazan crystals, and absorbance was measured at 570 nm in a plate reader.

Human THP-1 monocytes were cultured and maintained as described by Villalva et al. (39), with modifications reported by our group (31). For toxicity assay, cells were seeded in 24-well plates at a density of 5 × 10^5^ cells/ml, and monocytes were differentiated to macrophages by maintaining the cells with 100 ng/ml of PMA for 48 h. Then, theT33 extract was added to the wells at different concentrations (60 and 120 μg/ml), and the viability of the cells was evaluated by using the MTT assay as described above.

Furthermore, human SH-SY5Y cells were grown, maintained, and differentiated following the protocol proposed by de Medeiros et al. (40), with some modifications. Briefly, cells were maintained in DMEM/Ham's F12 1:1 supplemented with 100 U/ml penicillin, 100 μg/ml streptomycin, and 250 ng/ml antimycotic at 37°C in a humidified atmosphere of 5% of CO_2_. Only attached cells were maintained, and floating cells were discarded.

For neuronal differentiation, cells were treated with retinoic acid (RA) and brain-derived neurotrophic factor (human) (BDNF) for 7 days, as follows: first, the cells were plated and after 4 days, the cell culture medium was changed with a fresh medium with 10% FBS (day 0 of differentiation). Then, after 24 h (day 1), the cell culture medium was replaced with a fresh medium supplemented with 1% FBS and 10 μM of RA. After 3 days (day 4), the cell culture medium was replaced with a fresh medium with 1% FBS, 10 μM of RA, and 50 ng/ml BDNF. Finally, 3 days after (day 7), differentiated cells were seeded in 24-well plates at a density of 8 × 10^5^ cells/ml for 24 h. To evaluate the neurotoxic effect, the cells were incubated with different concentrations (60 and 120 μg/ml) of the T33 extract for 24 h, and the viability of the cells was tested by using the MTT assay as described above.

In all cases, the toxicity of the extracts is shown as relative cell viability, which is expressed as the percentage of living cells compared with controls (DMSO-treated). DMSO, used for diluting the extract, did not exceed a concentration of 0.4% (v/v). All the experiments were performed in triplicate.

### Liquid Chromatography-Tandem Mass Spectrometry (UHPLC-q-TOF-MS/MS) Analysis of Tamarillo Epicarp Extract

A liquid chromatography (Agilent 1290 UHPLC system; Agilent Technologies, Sta. Clara, CA, United States) coupled to a quadrupole-time-of-flight mass spectrometer (Agilent 6540 q-TOF MS; Agilent Technologies, Sta. Clara, CA, United States) by an orthogonal ESI source was used for the tentative identification of compounds present in the most active extract (T33). A 5-μl aliquot of the sample was injected at a flow rate of 0.5 ml/min in a Zorbax Eclipse Plus C18 column (2.1 × 100 mm, 1.8 μm particle diameter; Agilent Technologies, Santa Clara, CA, United States) at 30 °C, and the compounds were separated using a mobile phase consisting of A (water 0.01% formic acid) and B (acetonitrile 0.01% formic acid) in a gradient elution: 0 min, 0% B; 7 min, 30% B; 9 min, 80% B; 11 min, 100% B; 13 min, 100% B; 14 min, 0% B. The mass spectrometer was operated in negative mode, and MS and MS/MS analyses were performed for the structural analysis of all the compounds. MS parameters were the following: capillary voltage, 4000 V; nebulizer pressure, 40 psi; drying gas flow rate, 10 L/min; gas temperature, 350°C; skimmer voltage, 45 V; fragmentor voltage, 110 V. The MS and auto MS/MS modes were set to acquire m/z values ranging between 50 and 1,100 and 50 and 800, respectively, at a scan rate of 5 spectra per second. The Agilent Mass Hunter Qualitative Analysis software (B.08.00) was used for post-acquisition data processing, making use of filtering tools by fragments features based on diagnostic product ions (DPIs) and/or neutral loss of interest, or compound search by MS or MS/MS features that greatly enhance data interpretation. Peak identification in the TE extracts was performed by generation of the molecular formula with a mass accuracy of 5 ppm, and the characterization strategy was based on ESI-MS (accurate mass and isotopic distribution) and ESI-MS/MS (fragmentation pattern) data, together with information previously reported in the literature and databases, such as METLIN Metabolite Database (http://metlin.scripps.edu), Reaxys (https://www.reaxys.com), and PubChem (https://pubchem.ncbi.nlm.nih.gov/).

### Statistical Analysis

Experimental data results are given as mean ± SD. They were analyzed by ANOVA, and means were compared by Tukey's honestly significant difference (HSD) test (SPSS statics V15 IBM, New York, United States) at a 95% confidence level. Means labeled with different alphabetical letters in the same column of the table are considered statistically different at *p* < 0.05. Besides, a statistical test of correlation was performed in order to establish the degree of influence of the independent factors on the response variables and the interaction between them.

## Results and Discussion

### Pressurized Liquid Extraction Optimization

Considering the scarce information found in the literature about TE bioactivities and composition, the first goal of this study was to optimize PLE conditions using an RSM to test its efficiency toward the extraction of bioactive compounds. Based on results from Martin et al. (8), TE comprised high amounts of phenolic constituents and carotenoids. Wang and Zhu's review referred to the most relevant biological activities of tamarillo fruits and their by-products (7), and a mild AChE activity was reported by Hassan and Bakar for a methanolic extract from TE obtained by solid-liquid extraction (41).

Bearing this composition in mind, an experimental CCD was proposed in order to study the effect of the two main parameters involved in the extraction by PLE, i.e., extraction temperature (60–180°C) and green solvent composition (water/ethanol mixtures containing 2% formic acid), on the ability to obtain extracts with multitarget bioactivities. Selection of the range of the factors to be optimized was done according to previous results obtained by our research group targeting phenolic compounds (26). Moreover, 2% formic acid was employed in all solvent compositions as suggested by Brazdauskas et al. (27). A CCD at three levels was set up, as shown in Table 1, which also includes the different response variables studied. In this sense, as previously mentioned, AD is related to the accumulation of toxic amyloid peptides, oxidative stress, and neuroinflammation; therefore, those biological activities were selected as response variables and tested by using *in vitro* assays such as ABTS, LOX, and AChE. Moreover, other responses were also chosen to measure TPC, TFC, and TCC. All the responses were statistically analyzed, and a regression analysis was performed on the experimental data. The coefficients of the model(s) were evaluated for significance by using ANOVA for the different response variables (Supplementary Table 1).

**Table 1:** Experimental design and results of response variables [yield, total phenolic content (TPC), total flavonoid content (TFC), total carotenoid content (TCC), antioxidant (ABTS), anti-inflammatory (LOX), and anti-acetylcholinesterase (AChE) activities] for TE extracts obtained by pressurized liquid extraction (PLE).

**Sample**	**Solvent composition**	**Temp**	**Extraction yield**	**TPC**	**TFC**	**TCC**	**ABTS**	**LOX**	**AChE**
	**% EtOH:H_**2**_O (%v/v)**	**(°C)**	**(%)**	**(mg GAE/g extract)**	**(mg QE/g extract)**	**(mg CE/g extract)**	**IC_50_ (*μ*g/mL extract)**		
T11	0	60	43.01	19.190 ± 0.76	0.290 ± 0.04	11.471 ± 0.00	15.190 ± 0.01^e^	70.131 ± 0.06^b^	308.641 ± 3.52^f^
T12	0	120	64.98	20.640 ± 0.14	0.340 ± 0.13	0.860 ± 0.04	15.980 ± 0.20^e^	64.658 ± 0.34^b^	284.201 ± 2.03^f^
T13	0	180	75.85	58.480 ± 0.37	3.240 ± 0.44	46.081 ± 0.44	5.990 ± 0.04^b^	53.122 ± 0.20^b^	132.475 ± 0.17^cd^
T21	50	60	41.06	32.230 ± 0.35	0.410 ± 0.01	13.240 ± 0.31	15.710 ± 0.30^e^	185.951 ± 7.40^f^	253.907 ± 0.60^e^
T22	50	120	39.95	33.060 ± 0.52	0.790 ± 0.02	10.030 ± 0.34	15.690 ± 0.30^e^	111.328 ± 0.40^c^	251.487 ± 0.50^e^
T23	50	180	66.19	53.081 ± 0.00	2.040 ± 0.51	28.910 ± 0.67	6.720 ± 0.03^b^	58.213 ± 0.00^b^	152.761 ± 0.60^d^
T31	100	60	6.73	38.781 ± 0.08	2.760 ± 0.10	89.440 ± 0.01	13.510 ± 0.18^d^	145.401 ± 7.82^de^	236.699 ± 0.85^e^
T32	100	120	22.28	75.013 ± 0.28	3.000 ± 0.05	56.600 ± 0.63	12.370 ± 0.10^c^	161.261 ± 0.87^ef^	231.001 ± 2.81^e^
T33	100	180	36.25	92.090 ± 0.30	4.400 ± 0.19	107.152 ± 0.55	6.330 ± 0.01^b^	48.301 ± 0.61^b^	97.466 ± 0.82^b^
Galantamine^*^							–	–	0.400 ± 0.02^a^
Quercetin^*^							–	125.731 ± 0.73^cd^	–
Trolox^*^							2.500 ± 0.02^a^	–	–
Ascorbic acid^*^							25.000 ± 0.30^f^	–	–
Rosemary^**^							35.631 ± 0.14^g^	9.820 ± 0.88^a^	107.858 ± 0.39^bc^

*Results are expressed as the mean ± SD (n = 3). Different letter in the same column shows significant statistical differences (p < 0.05)*.

* *Chemical standard*.

** *Reference natural extract*.

As can be seen in Table 1, the response variables show different behavior depending on the extraction conditions. Considering their interest not only as a global composition but also in terms of “multitarget” bioactivity against AD, the responses will be individually analyzed and discussed in terms of the correlation observed among them and with the total content of phenolics, flavonoids, and carotenoids.

### Effect of Extraction Conditions on Yield and Total Bioactive Compound Content

**Figure 2:**
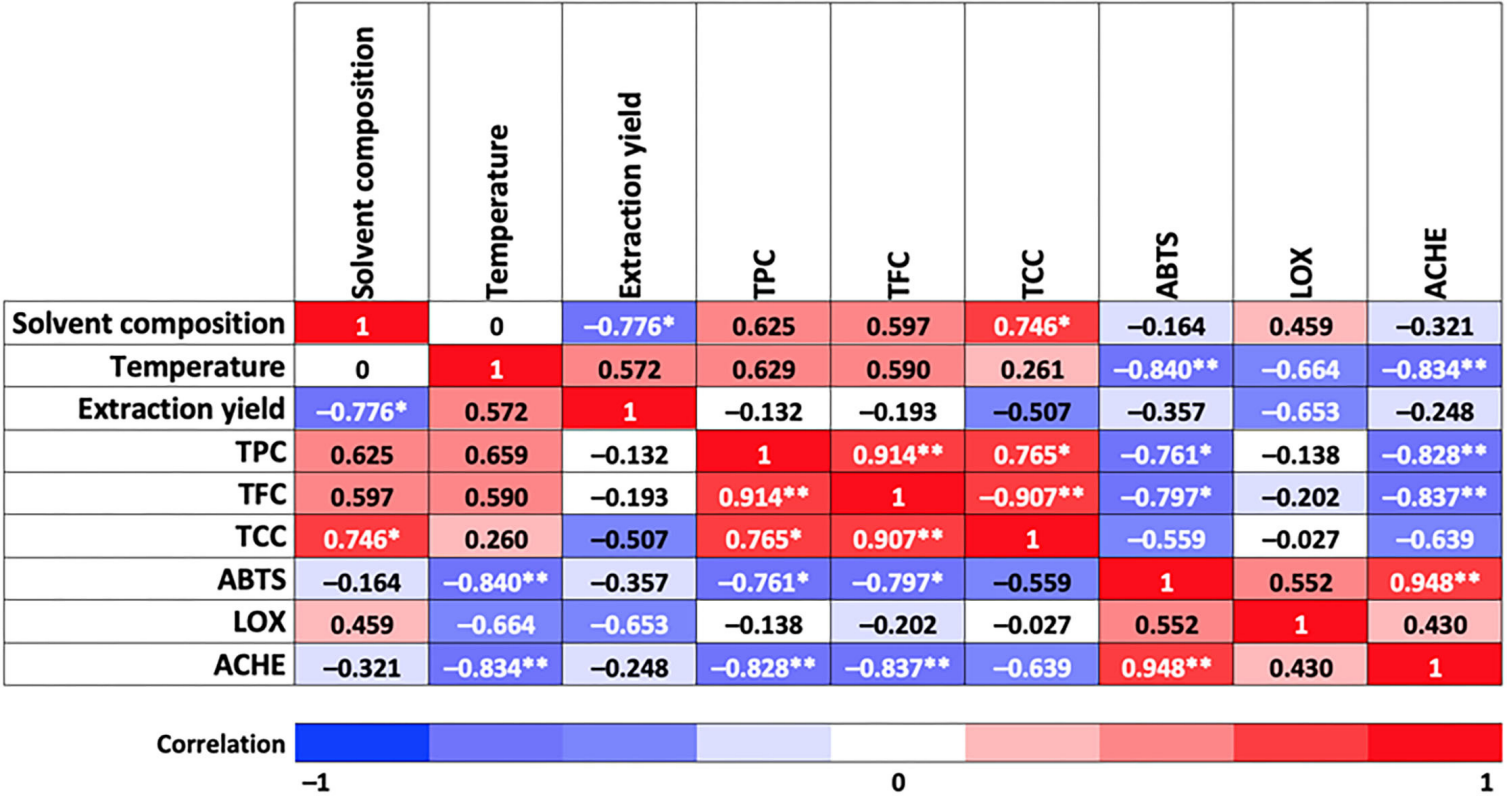
Correlation between independent factors and response variables involved in this study. Asterisks indicate a correlation at 0.05 significance level (*) and at 0.01 significance level (**).

Pearson's correlation model shows high (−0.78) and moderate (0.57) correlations between solvent composition, extraction temperature, and total yield, respectively, and as shown in Figure 2. Thus, the highest global yields of the PLE extracts were achieved with 100% water (98% water, 2% formic acid) and with water:ethanol (50:50) mixtures as extraction solvent. As a general trend, experimental data showed an increase in the global extraction yield by raising the extraction temperature from 60 to 180°C; this result was expected, considering that an increase in temperature improves mass transfer from the sample to the extraction solvent, increasing the solubility of the compounds and reducing solvent viscosity (thus favoring penetration of the solvent into the matrix).

As for TPC and TFC, responses follow the same trend as total yield, that is, increasing their value by increasing temperature within the same extraction solvent, although in this case, higher TPC and TFC were obtained using 100% EtOH (values equal to 92.09 mg GAE/g extract and 4.4 mg QE/g extract, respectively). As for TCC, the highest values were achieved at the highest and lowest temperatures for all the solvents tested, being also 100% ethanol the one providing the highest TCC (107.15 mg CE/g extract). A high (0.75) and moderate (0.63 and 0.60) correlations between TCC, TPC, and TFC, respectively, with solvent composition, were observed in Figure 2. Moreover, TPC and TFC showed a moderate correlation with extraction temperature (0.66 and 0.59, respectively). It is also interesting to observe correlations among the bioactive compounds measured; for instance, between TPC and TCC (high correlation, 0.77) and TPC and TCC with TFC (very high correlation, 0.91). A Pareto diagram for the effect of solvent composition and temperature on total bioactive compound content, and response surface plots are shown in Supplementary Figures 1, 2.

#### Effect of Extraction Conditions on ABTS, LOX, and AChE Activities. Correlation With TPC, TFC, and TCC

##### Antioxidant Activity

The analysis of variance test applied to determine the behavior of different extraction treatments in response to ABTS variable showed significant statistical differences compared with Trolox, ascorbic acid chemical standard, and rosemary natural standard, at *p* < 0.05 (see Table 1). However, it is remarkable that all the samples exhibited lower IC_50_ than ascorbic acid and rosemary, which means a high antioxidant activity of the TE extracts. Moreover, Figure 2 shows a very high correlation between ABTS and extraction temperature (−0.84) and almost no effect of the extraction solvent (−0.16). Therefore, higher antioxidant activity is expected at the highest extraction temperature (obtained extracts at 180°C exhibited near half IC_50_ values compared with those obtained at lower extraction temperatures), independently of the extraction solvent used. The behavior of the antioxidant activity as response variable as a function of temperature and solvent composition is presented in Supplementary Figures 3, 4.

In terms of chemical composition, a high correlation was observed between ABTS-TPC (−0.76) and ABTS-TFC (−0.8) (see Figure 2), which means that a lower IC_50_ value is achieved with a higher content of phenolic and flavonoid compounds. In fact, Table 1 shows that higher ABTS scavenging capacity was reached by the T13, T23, and T33 sample groups (IC_50_ = 5.99 ± 0.04, 6.72 ± 0.03, and 6.33 ± 0.01 μg/ml, respectively), and also reports the highest content of phenolic (58.48, 53.08, and 92.09 mg GAE/g extract) and flavonoid compounds (3.24, 2.04, and 4.4 mg QE/g extract). Skinner et al. (42) mentioned that tamarillo skin is the highest contributor to the phenolic content (by weight) compared with pulp and seeds, being hydroxycinnamic acid (HCA) derivatives the major compounds. The observed antioxidant activity could be attributed mainly to phenolic acids identified in this study, such as HCA group, quinic acid, caffeoyl glucoside, chlorogenic acids (CGAs), and rosmarinic acids, among others, which is in agreement with previously reported results (6, 7, 43).

In the case of flavonoids, a higher amount of these bioactive metabolites has been reported in tamarillo peel (3.36 mg rutin eq/g d.w.) compared with pulp (2.41 mg rutin eq/g d.w.) by Mutalib et al. (44). These authors also stated that flavonoid content is 3-fold larger in ethanolic extracts than in water extracts. This is in agreement with our results in which a higher flavonoid content was achieved in the T31, T32, and T33 samples (2.76, 3, and 4.4 mg QE/g extract, respectively), extracted with 100% EtOH. The antioxidant activity of flavonoids is attributed to the presence of several -OH groups in the molecule; in this sense, some studies reported the strong antioxidant and radical scavenging capacity of these phytochemical compounds (45–47). According to Nijveldt et al., flavonoids exhibit strong cell protection against ROS through different working mechanisms including an increase in the function of endogenous antioxidants (46). First, flavonoids can work as direct radical scavengers being oxidized by radicals because of the high reactivity of the OH group of the flavonoids; hence a radical molecule becomes more stable and inactive; an example of these are rutin (48), luteolin, and quercetin (49, 50); all of them are found in this study. Second, NO^·^ plays a significant role in molecular events involved in neurodegenerative diseases, promoting neuronal death through oxidative damage on cellular lipids, proteins, and ADN (51); therefore, flavonoids are able to remove the overconcentration of NO^·^ radicals (46, 52, 53). Third, other antioxidant pathways attributed to flavonoids are inhibition or xanthine-oxidase (source of highly toxic hydroxyl radicals) enzymes or iron-chelating capacity. The key role of quercetin (one of the most powerful bioflavonoids) as an inhibitor of NO synthase (53), xanthine oxidase (54), and iron-chelator (55) is noticeable.

Total carotenoid content exhibited a moderate correlation with ABTS (−0.56) (Figure 2). In this study, an important content of total carotenoids (89.44, 56.6, and 107.15 mg CE/g extract) was achieved in the ethanolic extracts (T31, T32, and T33) at 60, 120, and 180°C, respectively, being T33 the extract with the highest TCC (107.15 ± 2.55 mg CE/g extract) and the highest ABTS scavenging capacity (IC_50_ = 6.33 ± 0.01 μg/ml, see Table 1). In fact, tamarillo fruits are classified according to skin color, which is related to the presence of carotenoids. According to this study, a higher amount of carotenoids was found in TE than in the results reported by Ghosh et al. (56) for peels from orange, mango, and pomegranate grape. Likewise, the presence of vitamin C (ascorbic acid) in tamarillo is also important not only for its recognized contribution to antioxidant activity but also when synergic interaction within the metabolic pathways of other vitamins is considered, despite its smaller proportion in the peel than pulp (15–30% lower) according to results reported by Diep et al. (57).

Furthermore, vitamin E (α-tocopherol form) is another relevant antioxidant present in TE with scavenger capacity of lipid peroxyl radicals and potent agent against oxidative damage involving chronic diseases, such as cardiovascular diseases, cancer, and AD (31, 37, 58–60). According to Diep et al. (57), a higher content of α-tocopherol was found in the peel than in the pulp of all studied tamarillo varieties, which undoubtedly contributes to an increase in important results in terms of the antioxidant potential of tamarillo epicarp extracts.

##### Anti-inflammatory Activity

Neuroinflammation is the mechanism of the central nervous system that occurs in response to trauma, infections, or neurodegenerative diseases. Considering the importance of anti-inflammatory activity on the multitargeted approach of this study, this bioactivity was measured in all the extracts through the enzymatic activity of LOX (data shown in Table 1).

The ANOVA showed no significant differences (*p*- < 0.05) among the T11, T12, T13, T23, and T33 samples. Nevertheless, there is a statistically significant difference between the mentioned samples and quercetin (employed as chemical standard) (IC_50_ = 125.73 μg/ml) having, on average, an IC_50_ twice the one obtained for T11, T12, T13, T23, and T33 (70.13, 64.65, 53.12, 58.21, and 48.3 μg/ml, respectively), suggesting a high anti-inflammatory potential of the TE extracts. Regarding correlations between factors and responses involved in LOX, a medium-high correlation of LOX with extraction yield and extraction temperature (−0.65 in both cases) and with extraction solvent (0.46) was observed (Figure 2). Notwithstanding, the best LOX values (lowest IC_50_) were achieved at the highest temperature (180°C) for all the extracting solvents studied, with IC_50_ of 53.12 ± 2.2, 58.21 ± 3, and 48.3 ± 1.61 μg/ml the for T13, T23, and T33 extracts, respectively.

A moderate correlation (0.55) with ABTS is also observed (see Figure 2). It means that the TE extracts that showed a potential antioxidant activity could also have a potential anti-inflammatory response, as can be seen in T13, T23, and T33.

The release of arachidonic acid is a starting point for a general inflammatory response (46). Hence, certain flavonoids act by decreasing the level of the arachidonic acid pathway, prostaglandins, leukotrienes, and NO, recognized mediators in inflammation through the inhibition of enzymes involved in the process, such as phospholipase A_2_ (PLA_2_), cycloxygenase (COX), LOX, and NO synthase (45, 51). As a consequence, the better potential of LOX inhibitory capacity was achieved for the T13, T23, and T33 extracts characterized by the higher content of flavonoids with recognized anti-enzymatic effect (47, 61). In this sense, quercetin, a flavonol used in this study, is the standard generally employed in anti-inflammatory assays.

Our results are in agreement with those recently published by Li and Li (61) who evaluated the inhibitory activity of tamarillo peel extracts against inflammatory markers (PGE-2, IL-1β, and TNF-α) on LPS-induced macrophages (RAW 264.7 cell line). The authors concluded that low concentrations of tamarillo skin extracts (12.5 and 25 μg/ml) exerted anti-inflammatory activity by reducing these markers, attributing this activity to the presence of high content of flavones, isoflavones, and flavonols.

##### Anti-cholinergic Activity

As shown in Table 1, the IC_50_ values represent the concentration (μg/ml) of the TE extract capable of producing half inhibition of AChE enzyme activity; thus, lower IC_50_ means greater enzymatic inhibition capacity. Through the analysis of variance (Supplementary Table 1), the behavior of the different treatments applied to TE on the response variable AChE was studied. As can be seen, ANOVA and Tukey *post hoc* multiple comparisons determined that all the analyzed extracts showed significant differences compared with galantamine as chemical standard, and T13, T23, and T33 exert a significant influence on the AChE response variable, with IC_50_ values of 132.47 ± 5.17, 152.76 ± 10.6, and 97.46 ± 6.82 μg/ml extract, respectively. These values allow them to be classified as within moderate potency AChE inhibitors, with a range of 20 < IC_50_ <200 μg/ml, according to data reported by Dos Santos et al. (62) in the general classification of natural extracts efficacy.

As a summary, these results suggested that when the TE (yellow ecotype) is subjected to the highest tested extraction temperature (180 °C) by PLE, it is possible to obtain extracts rich in flavonoid, phenolic, and carotenoid-type compounds, showing promising anti-AChE, anti-inflammatory, and antioxidant activities.

##### Effect of Extraction Conditions on Total Bioactive Constituent Contents and Biological Activities. Multiple Response Optimization

**Figure 3:**
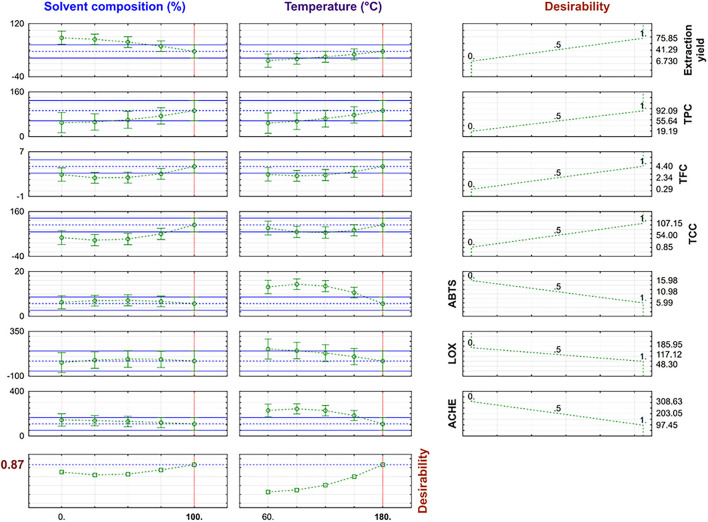
Desirability value and predicted response variables in multi-response optimization.

Due to the promising results obtained for the TE extracts under PLE conditions, an optimization of the operating conditions considering all responses studied was performed to achieve an extract with potential for a “multitarget” therapy of AD (antioxidant, anti-inflammatory, and anti-cholinergic); to do this, a desirability function was calculated. Profiles for predicted values estimated by desirability function are shown in Figure 3.

The optimal conditions were 100% EtOH and 180°C with a 0.87 desirability value, corresponding to the extraction conditions of the T33 extract. The desirability value was close to 1, and the predicted values obtained by the global desirability function under optimum conditions were compared with experiment T33 (Table 2). The observed and predicted data were within the confidence intervals (Figure 3) and, as can be seen, the proximity between predicted and experimental data confirmed that the selected RSM model was successfully applied for obtaining a TE extract with anti-neurodegenerative potential.

**Table 2:** Experimental and predicted values for all response variables at 100% EtOH and 180°C.

**Responses**	**Experimental (T33 extract)**	**Predicted**	**−95%Cl**	**+95%Cl**
Extraction yield (%)	36.25	36.32	26.40	46.24
TPC (mg GAE/g extract)	92.09 ± 0.30	93.05	76.72	109.38
TFC (mg QE/g extract)	4.40 ± 0.19	4.37	3.16	5.55
TCC (mg CE/g extract)	107.15 ± 2.55	100.47	89.57	111.34
ABTS (IC_50_, μg/mL)	6.33 ± 0.01	5.65	2.67	8.59
LOX (IC_50_, μg/mL)	48.30 ± 1.61	51.68	49.98	53.36
AChE (IC_50_, μg/mL)	97.46 ± 6.82	108.67	102.16	115.18

As a summary, conditions selected for obtaining the T33 extract provided a medium yield (36.25%), and the highest total content of phenolics (92.09 mg GAE/g extract), flavonoids (4.4 mg QE/g extract), and carotenoids (107.15 mg CE/g extract), and the lowest IC_50_ values (ABTS: 6.33 μg/ml; LOX: 48.3 μg/ml; AChE: 97.46 μg/ml). In order to deepen the knowledge of the T33 extract and its ability as a multitarget extract against AD, complementary biological activities (ROS, RNS, BChE) and chemical characterization by UHPLC-q-TOF-MS/MS were performed.

### ROS, RNS, and BChE Activities

Table 3 shows the results obtained on the complementary biological activities related to neuroprotective potential tested on the T33 extract. Considering that one of the main symptoms of neurodegenerative disorders is oxidative and nitrosative stress caused by an imbalance between the production of ROS/RNS and response capacity with antioxidant molecules (generating as a consequence neuronal damage and cell death), ROS and NRS activities were measured for the T33 extract. As can be seen in Table 3, the T33 extract exhibited an IC_50_ value lower than those given by rosemary extract (IC_50_ = 2.54 ± 0.14 μg/mL against IC_50_= 4.51 ± 0.23, respectively), requiring almost half concentration than rosemary extract to reach the same antioxidant capacity, demonstrating a higher ROS scavenging property but lower than Trolox standard (IC_50_= 1.29 ± 0.09 μg/ml). In terms of RNS, the IC_50_ value of the T33 extract improved almost twice compared with ascorbic acid standard (IC_50_ = 599 ± 5.92 μg/ml against IC_50_ = 1,100.9 ± 13.96 μg/ml, respectively), but it was less effective than the rosemary standard (IC_50_ = 95.35 ± 6.45 μg/ml). As mentioned previously, the presence of phenolic and flavonoid-type metabolites with free radical scavenging and inhibitory enzymatic properties, such as quercetin, luteolin, and rutin, exerts powerful protection against ROS and RNS. As for anti-BChE inhibitory activity, the T33 extract exhibited significant differences (*p* < 0.05) compared with both standards studied (galantamine and rosemary). However, better IC_50_ (85.462 ± 0.68 μg/ml) was achieved by the TE extract than those reached by the orange by-product ethanolic extract obtained by maceration (IC_50_ = 494 ± 68 μg/ml) and reported by Sánchez-Martínez et al. (36).

**Table 3:** IC_50_ values (μg/ml) of reactive oxygen species (ROS)-oxygen radical absorbance capacity (ORAC), reactive nitrogen species (RNS), and anti-butyrylcholinesterase enzyme (BChE) assays for T33 tamarillo epicarp extract.

**Sample**	**ROS**	**RNS**	**BChE**
T33	2.540 ± 0.14^b^	599.005 ± 0.92^b^	85.462 ± 0.68^c^
Galantamine^*^	–	–	0.400 ± 0.02^a^
Ascorbic acid^*^	–	1100.901 ± 3.96^c^	–
Trolox^*^	1.290 ± 0.09^a^	–	–
Rosemary (SFE)^**^	4.510 ± 0.23^c^	95.356 ± 0.45^a^	54.891 ± 0.53^b^

*Results are expressed as the mean ± SD (n = 3)*.

* *Chemical standard*.

** *Reference natural extract*.

*Different letter in the same column shows significant statistical differences (p < 0.05)*.

Therefore, results on the complementary biological activities (ROS, RNS, and BChE) obtained for the T33 extract allowed to confirm its important neuroprotective potential.

### UHPLC-q-TOF-MS/MS Analysis

**Figure 4:**
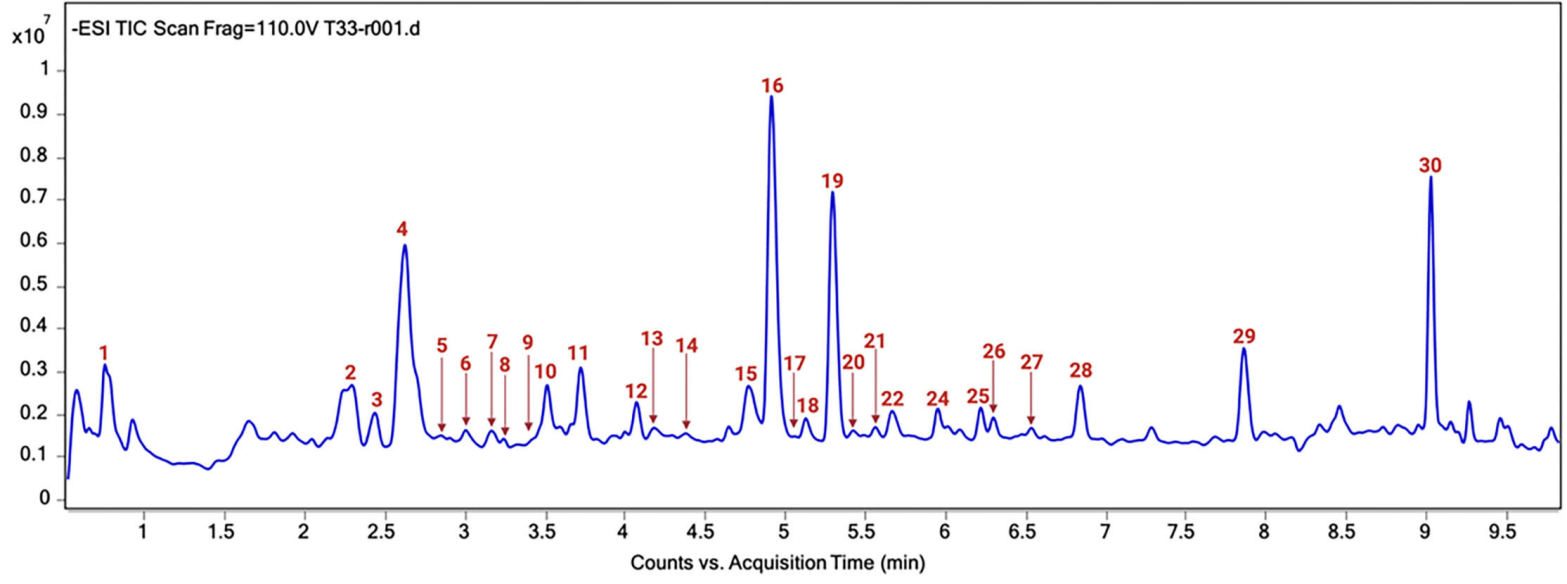
Total ion current (TIC) chromatogram of T33 extract. Peak identification can be found in Table 4.

The chromatographic profile of the T33 extract is shown in Figure 4 and the tentatively identified compounds are summarized in Table 4, namely, the retention time (min), molecular formula, experimental and theoretical *m/z*, error (ppm), and main MS/MS fragments. A total of 30 compounds were tentatively identified belonging to different chemical classes, namely, hydroxybenzoic acids and derivatives, HCAs, CGAs and derivatives, acetyl quinic acid derivatives, and flavonoids, among others. According to previous studies, neutral losses of H_2_O (18 Da) or CO_2_ (44 Da) molecules were frequently found for phenolic acids and derivatives, such as compounds 1 (quinic acid), 7 (hydroxybenzoic acid), 8 (gallic acid), 14 (caffeic acid), 26 (dihydrocaffeic acid), 23 (sinapic acid), and 13 (syringaldehyde) (63, 67, 69, 76). However, compound 9 showed a different fragmentation pattern including the main ion fragment at *m/z* 93 because of loss of C_3_H_2_O_2_ (70 Da); hence, the structure was tentatively assigned to *p*-coumaric acid. Phenolic glycosides were detected by the characteristic direct loss of the hexose moiety, such as syringic acid hexoside (compounds 2 and 3) and caffeoyl hexoside (compound 6), as well as by the fragmentation of these compounds yielding aglycone ions (64, 66). CGAs were distinguished on the basis of their typical fragmentation patterns, owing to the loss of phenolic acid residues; these compounds included caffeoylquinic acid (compounds 5, 10, and 12) and caffeoylshikimic acid (compounds 15 and 17). Caffeoylquinic acid isomers showed a fragment ion at *m/z* 191 because of loss of caffeoyl moiety, and 173 caused by consecutive loss of H_2_O; also, fragment ions at *m/z* 179 and 161 were observed because of loss of quinoyl moiety and subsequent H_2_O loss (63). Similarly, caffeoylshikimic acid isomers showed fragments at *m/z* 179, 161, and 135 corresponding to the losses of shykimoyl, shykimoyl, and H_2_O, and shykimoyl and CO_2_ moieties, respectively (64). Compound 25 was tentatively identified as rosmarinic acid since the MS/MS fragmentation of its pseudomolecular peak ([M – H]^−^ion at *m/z* 359) led to three peaks at *m/z* 197, 179, and 161 corresponding to the deprotonated form of 3-(3,4-dihydroxy-phenyl) lactic and caffeic acids and their dehydrated forms, respectively, which have been previously described (72, 73). Quinic acid derivatives were also observed in the T33 extract. Compounds 11, 16, and 19 were tentatively identified as acetyl quinic acid and acetyl quinic acid derivatives 1 and 2, respectively, because of the loss of acetyl moiety resulting in the quinic acid group observed at *m/z* 191 in MS/MS experiments (68). Compounds 16 and 19 were the most intense peaks in the chromatogram along with 4 and 30 (see Figure 4). Compound 4 was tentatively assigned to ethyl citrate, which gave a [M – H]^−^ion at *m/z* 219 with fragment ion at *m/z* 111 in the MS/MS experiment caused by the loss of ethyl acetyl and two water groups (65). Compound 30 was identified as phytuberin, a sesquiterpenoid found in other Solanaceae species (77), which showed a [M – H]^−^ion at *m/z* 293 in the ESI-MS mode and daughter MS/MS ions at *m/z* 236, 221, and 192, indicating the losses of acetate, acetate, and CH_3_, and isopropyl acetate groups, respectively, as described by Serralheiro et al. (75). Methyl esters of CGAs and methyl quinates were also found in the T33 extract. These compounds are widely distributed in plant materials and frequently appear as extraction artifacts in these samples (70). Compounds 18, 22, 24, and 27 were tentatively assigned to methyl caffeoyl quinate, methyl feruloyl quinate isomer 1, methyl feruloyl quinate isomer 2, and methyl feruloyl hexoside, respectively. The fragmentation behavior of methyl esters of CGAs and methyl quinates showed the loss of methyl quinate moiety and subsequent losses of CH_3_, H_2_O, or CO_2_, as can be seen for the explained MS/MS product ions in Table 4. In the case of methyl feruloyl hexoside, direct loss of the hexose (fragment ion at *m/z* 207) allowed identification. Compound 29 was identified as ethyl caffeate, which gave a characteristic fragment ion at m/z 161 because of the loss of ethanol. Ethyl caffeate is presumably a product of trans-esterification of caffeic acid esters and ethanol, formed during the PLE extraction process. Barth et al. (74) have explained this phenomenon considering that esterification is a reaction that occurs between a carboxylic acid and an alcohol, being favored by the increase in temperature, through nucleophilic substitution on acyl carbon in which the nucleophile (ethanol) attacks the carbon of carbonyl group (caffeic acid), leading to the formation of the ester. Regarding flavonoids, compounds 20 and 21 were tentatively assigned to rutin and quercetin hexoside, respectively, which were distinguished on the basis of their typical loss of sugar moiety (rutinose or hexose) resulting in the fragment ions observed at *m/z* 301 (71). HCA and CGA derivatives, as well as flavonoids, have been described as the major types of phenolic compounds in the golden-yellow tamarillo fruit (7). Orqueda et al. (16) identified several caffeic acid derivatives (i.e., caffeoyl hexoside, caffeoylquinic acid isomers, among others) and related phenolics, rosmarinic acid derivatives, and flavonoids (i.e., quercetin hexoside, runtin, among others) by HPLC–ESI-MS/MS analysis on different segments of tamarillo fruit (Argentinian cultivar), such as the peel. Espin et al. (78) also identified and quantified some phenolic compounds from tamarillo fruit (Ecuatorian cultivar) finding a concentration that ranged between 1.27 and 42.73 mg caffeoylquinic acid/100 g dw and 12.22 and 32.85 mg rosmarinic acid/100 g dw. Loizzo et al. (79) reported the phenolic content in an ethanolic extract of tamarillo peel fruit (Colombian cultivar): chlorogenic acid (253.8 mg/kg extract), p-coumaric acid (0.2 mg/kg extract), sinapic acid (10.3 mg/kg extract), and syringaldehyde (0.7 mg/kg extract).

**Table 4:** Identification of chemical constituents in the T33 extract by liquid chromatography-tandem mass spectrometry (UHPLC-q-TOF-MS/MS).

**Peak**	***t*_**R**_ (min)**	**Tentative identified compound**	**Formula**	**Deprotonated molecular ion [M – H]^−^ (*m/z*)**		**Error (Δ ppm)**	**MS^**2**^ product ions (*m/z*)**	**References**
				**Measured**	**Theoretical**			
1	0.594	Quinic acid	C_7_H_12_O_6_	191.0567	191.0561	3.08	173 [M – H –H_2_O]^−^	(63)
2	2.304	Syringic acid hexoside isomer I	C_15_H_20_O_10_	359.1002	359.0984	5.09	197 [M – H – hexose]^−^, 161 [M – H – hexose – 2H_2_O]^−^	(64)
3	2.439	Syringic acid hexoside isomer II	C_15_H_20_O_10_	359.1001	359.0984	4.82	197 [M – H – hexose]^−^, 161 [M – H – hexose – 2H_2_O]^−^	(64)
4	2.625	1-Ethyl citrate	C_8_H_12_O_7_	219.0521	219.0510	4.90	111 [M –H – ethyl acetyl – 2H_2_O]^−^	(65)
5	2.821	4-*O*-Caffeoylquinic acid (cryptochlorogenic acid)	C_16_H_18_O_9_	353.088	353.0878	0.55	191 [M – H – caffeoyl]^−^, 179 [M – H – quinoyl]^−^, 161 [M – H – quinoyl – H_2_O]^−^	(63)
6	2.986	Caffeoyl hexoside	C_15_H_18_O_9_	341.0883	341.0878	1.45	179 [M – H –hexose]^−^	(66)
7	3.175	Hydroxybenzoic acid	C_7_H_6_O_3_	137.025	137.0244	4.20	93 [M – H –CO_2_]^−^	
8	3.211	Gallic acid	C_7_H_6_O_5_	169.0133	169.0142	−5.60	151 [M – H – H_2_O]^−^, 125 [M – H – CO_2_]^−^	(67)
9	3.470	4-Hydroxycinnamic acid (*p*-coumaric acid)	C_9_H_8_O_3_	163.0404	163.0401	2.04	93 [M – H – C_3_H_2_O_2_]^−^	
10	3.507	3-*O*-Caffeoylquinic acid (chlorogenic acid)	C_16_H_18_O_9_	353.089	353.0878	3.38	191 [M – H – caffeoyl]^−^, 161[M – H – quinoyl – H_2_O]^−^	(63)
11	3.702	Acetyl quinic acid	C_9_H_14_O_7_	233.0672	233.0667	2.24	191 [M – H – acetyl]^−^	(68)
12	4.054	5-*O*-Caffeoylquinic acid (neochlorogenic acid)	C_16_H_18_O_9_	353.0885	353.0878	1.97	191 [M – H – caffeoyl]^−^, 173 [M – H – caffeoyl – H_2_O]^−^, 161 [M – H – quinoyl – H_2_O]^−^	(63)
13	4.295	Syringaldehyde	C_9_H_10_O_4_	181.0511	181.0506	2.58	163 [M – H – H_2_O]^−^	
14	4.352	Caffeic acid	C_9_H_8_O_4_	179.0359	179.0350	4.89	135 [M – H – CO_2_]^−^	(69)
15	4.849	Caffeoylshikimic acid	C_16_H_16_O_8_	335.0779	335.0772	1.96	179 [M – H – shikimoyl]^−^, 135 [M – H – shikimoyl – CO_2_]^−^	(64)
16	4.908	Acetyl quinic acid derivative	C_10_H_16_O_7_	247.0834	247.0823	4.33	191 [M – H – acetyl]^−^	
17	4.984	Caffeoylshikimic acid	C_16_H_16_O_8_	335.0774	335.0772	0.47	179 [M – H – shikimoyl]^−^, 161 [M – H – shikimoyl – H_2_O]^−^	(64)
18	5.099	Methyl caffeoyl quinate	C_17_H_20_O_9_	367.1035	367.1034	0.21	191 [M – H – caffeoyl – CH_3_]^−^, 179 [M – H – methylquinate]^−^, 161 [M – H – methylquinate – H_2_O]^−^, 135 [M – H – methylquinate – CO_2_]^−^	(70)
19	5.293	Acetyl quinic acid derivative	C_10_H_16_O_7_	247.0825	247.0823	0.69	191 [M – H – acetyl]^−^	
20	5.399	Rutin	C_27_H_30_O_16_	609.1466	609.1461	0.81	301 [M – H – rutinoside]^−^, 300 [M – H – rutinoside – H]^−^	(71)
21	5.578	Quercetin hexoside	C_21_H_20_O_12_	463.0879	463.0877	0.54	301 [M – H – hexoside]^−^, 300 [M – H – hexoside – H]^−^, 271 [M – hexoside – CH_2_O]^−^, 255 [M – hexoside – CH_2_O – CO + H_2_O]^−^	(71)
22	5.693	Methyl feruloyl quinate isomer I	C_18_H_22_O_9_	381.1186	381.1191	−1.37	161 [M – H – methylquinate – CH_3_– H_2_O]^−^, 135 [M – H – methylquinate – CH_3_– CO_2_]^−^	(70)
23	5.819	Sinapic acid	C_11_H_12_O_5_	223.0607	223.0612	−2.23	179 [M –H – CO_2_]^−^	(76)
24	5.935	Methyl feruloyl quinate isomer II	C_18_H_22_O_9_	381.1202	381.1191	2.82	179 [M –H – methylquinate – CH_3_]^−^, 161 [M – H – methylquinate – CH_3_– H_2_O]^−^, 135 [M – H – methylquinate – CH_3_– CO_2_]^−^	(70)
25	6.171	Rosmarinic acid	C_18_H_16_O_8_	359.0783	359.0772	2.95	197 [M –H – caffeoyl]^−^, 179 [M – H – hydrocaffeoyl^−^, 161 [M – H – caffeoyl – H_2_O]^−^, 135 [M – H – hydrocaffeoyl – CO_2_]^−^	(72, 73)
26	6.416	Dihydrocaffeic acid	C_9_H_10_O_4_	181.0505	181.0506	−0.73	137 [M – H – CO_2_]^−^	(69)
27	6.498	Methyl feruloyl hexoside	C_17_H_22_O_9_	369.1191	369.1191	0.01	207 [M – H – hexose]^−^	
28	6.808	Not identified	C_41_H_72_O_10_	723.5037	723.5053	−2.18		
29	7.837	Ethyl caffeate	C_11_H_12_O_4_	207.0668	207.0663	2.30	161 [M – H – C_2_H_6_O]^−^, 133 [M – H – C_3_H_5_O_2_]^−^	(74)
30	9.051	Phytuberin	C_17_H_26_O_4_	293.1773	293.1758	5.00	236 [M – H – acetate]^−^, 221 [M – H – acetate – CH_3_]^−^, 192 [M – H –isopropyl acetate]^−^	(75)

According to Table 4, the UHPLC-q-TOF-MS/MS analysis of the T33 extract revealed an important group of phenolic acids, such as 4-hydroxycinnamic (9), caffeic (14), rosmarinic (25), and chlorogenic isomer (10, 12) acids, as well as quercetin hexoside (21) and rutin (20) into the flavonoid group that could play an important role in AChE inhibition. In fact, the contribution to the enzyme inhibitory activity attributed to rosmarinic acid (25, 80, 81) and the neuroprotective effect exerted by quercetin, among others (45–47) are widely recognized. According to Dos Santos et al. (62) flavonoids and phenolic compounds seem to act as noncompetitive inhibitors that bind to the peripheral anionic active site of AChE, and they highlight the potential inhibitory effect of quercetin. A study by Oboh et al. (82) suggested that the potential cholinergic enzymatic inhibition of phenolic compounds is a function of the number and position of their OH groups, which forms hydrogen bonds with specific amino acids at the active sites of the enzymes. These authors studied the inhibitory enzymatic activity of caffeic acid and CGAs separately, and they found that caffeic acid exerts higher anti-AChE and anti-BChE capacities than CGA. However, the inhibitory capacity of AChE was decreased when both phenolic acids interact together as a result of the antagonistic effect of chlorogenic on caffeic acid due to the presence of quinic acid moiety on the chlorogenic structure, that could affect the interaction of the phenolic acids with the active site of both AChE and BChE, causing a significant reduction in their enzyme inhibitory activity. The differences between the values reported by some authors and those in this study can be attributed mainly to the tamarillo variety studied as well as the technique and extraction parameters applied. Regarding anti-inflammatory activity, ethyl caffeate (29) has been reported as a bioactive compound with anti-inflammatory properties (74), in addition to quercetin and rutin.

### Cytotoxicity and Neurotoxicity Study

**Figure 5:**
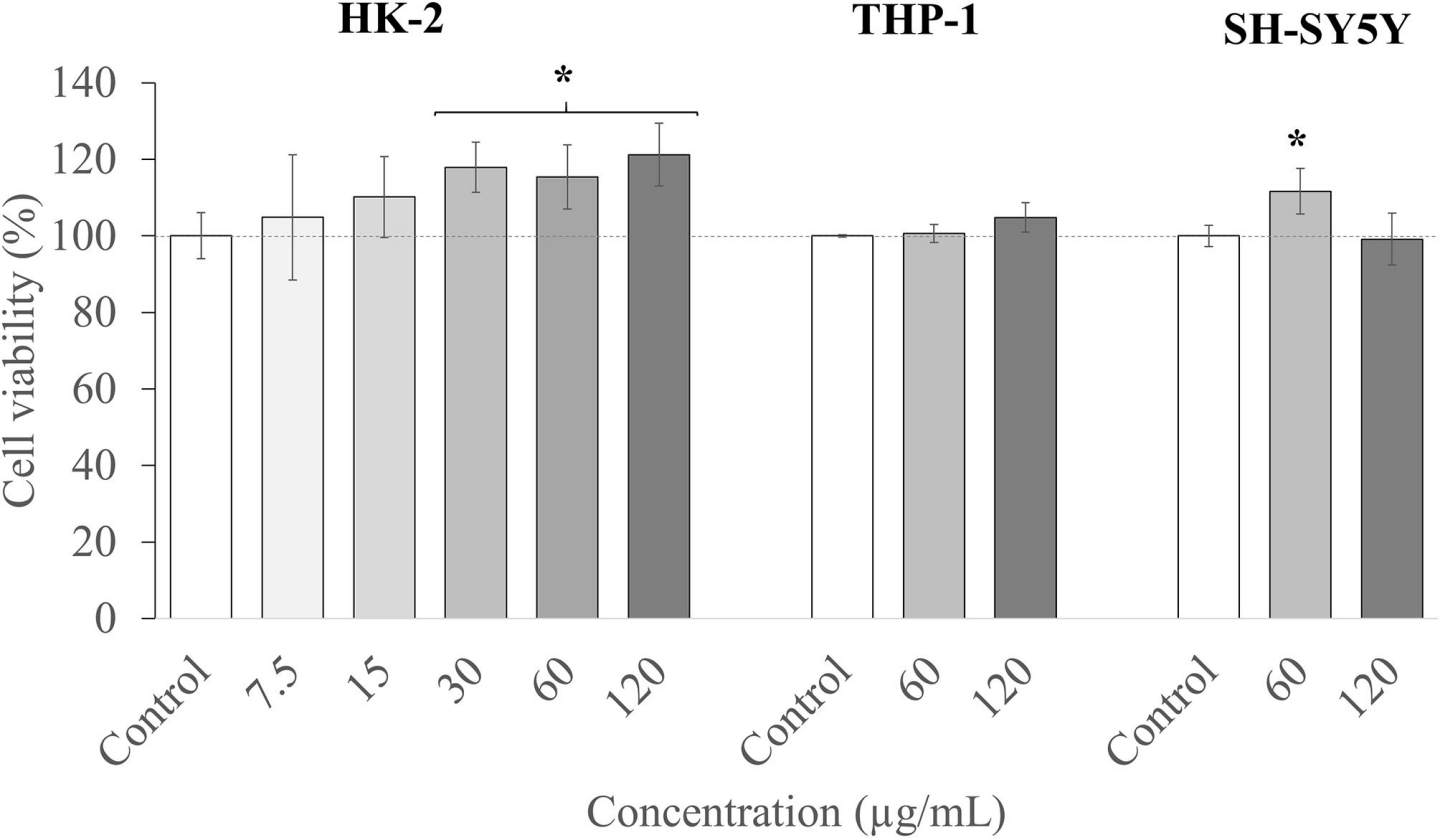
Cell viability in human kidney-2 (HK-2), THP-1, and SH-S5Y5 cell lines upon treatment for 24 h with different concentrations of T33 extract. Error bars are given as the SD of three independent experiments. (*) indicates significant differences between the treated and control samples, *p* < 0.05.

Among standard cell lines, human kidney 2 (HK-2) cells are widely used as a model to predict *in vitro* toxicity in humans (83), so HK-2 cells were chosen in this article in order to study the toxicity effect of the T33 extract as a first step. As it can be observed in Figure 5, the T33 extract presented no toxicity at any concentration tested (7.5 to 120 μg/ml) and even showed an increase in cell viability at the highest T33 concentrations.

Thereafter, the two highest nontoxic concentrations (60 and 120 μg/ml) of the extract were selected, and their cytotoxicity was evaluated in two specific cell lines. First, THP-1 cells were used, which are commonly employed to investigate the function and regulation of monocytes and macrophages in the cardiovascular system, and to study functions, mechanisms, and bioactivities of food compounds or pharmacological activities of drugs, among others (84). Besides, and in order to predict any neurotoxic effect of the extract, the neuroblastoma SH-SY5Y cell line was also employed. This model is widely used in *in vitro* studies of neurological disorders, such as AD, ischemia, or neurotoxicity (85). Moreover, these cells can be differentiated and acquire mature neuron-like features, thus becoming phenotypically closer to primary neurons (40).

As can be observed in Figure 5, the two concentrations tested (60 and 120 μg/ml) maintained the cell viability at the maximum level. To summarize, the T33 extract showed no toxicity (up to 120 μl) for all the tested cell culture lines, and further *in vitro* and *in vivo* studies could be performed to investigate its potential bioactivity. It should also be highlighted that the concentrations tested are relatively high compared with other matrices also tested in these models, such as macroalgae *Cystoseira usneoides* (86), or *Thymbra capitata* and *Thymus* Species (87), with toxicity reported in THP-1 cells at concentrations above 8 μg/ml.

## Conclusions

This study reported the potential of the tamarillo (*C. betacea*) epicarp as a promising source of bioactive extracts. TE extracts obtained by PLE have demonstrated, for the first time, their antioxidant, anti-inflammatory, and anti-cholinergic inhibitory activities. These extracts showed high phenolic, flavonoid, and carotenoid contents. The most active extract showed no toxicity in all the tested cell lines. The analysis of the optimum TE extract by UHPLC-q-TOF-MS/MS showed an important presence of hydroxycinnamic acid derivatives and other phenolic acids as well as quercetin hexoside and rutin as bioactive metabolites. Therefore, the results suggest that the TE subjected to PLE could become a promising natural by-product with multitarget activity against AD. This study attempts to approach sustainability and improvement of the quality of life from different perspectives. On one hand, the reduction of food waste through the development of environmentally friendly technologies is able to achieve improved and high added value extracts; on the other hand, the possibility of designing extracts with multitarget activity against important diseases, such as AD. The obtained results corroborate the interest of both, TE as a by-product and PLE as technology, to achieve promising extracts with confirmed bioactivities and composition, and represent a step forward toward the development of therapies based on natural products.

## Data Availability Statement

The datasets presented in this study can be found in online repositories. The names of the repository/repositories and accession number(s) can be found below: EBI MetaboLites, accession no: MTBLS3489.

## Author Contributions

ZS-M, FP-A, EI, and AC designed the experiments. ZS-M, FP-A, AV, RG, and JS-M performed the experiments. ZS-M and FP-A wrote the original draft. ZS-M, FP-A, DB-V, AV, and RG analyzed the data. EI and AC acquired funding and reviewed and edited the article. All authors contributed to the article and approved the submitted version.

## Funding

This research was funded by the Ministry of Economy and Competitiveness (MINECO), Spain, project AGL2017-89417-R and PID2020-113050RB-I00.

## Conflict of Interest

The authors declare that the research was conducted in the absence of any commercial or financial relationships that could be construed as a potential conflict of interest.

## Publisher's Note

All claims expressed in this article are solely those of the authors and do not necessarily represent those of their affiliated organizations, or those of the publisher, the editors and the reviewers. Any product that may be evaluated in this article, or claim that may be made by its manufacturer, is not guaranteed or endorsed by the publisher.
