# Supplementary material for: Neuroprotective Potential of Tamarillo (Cyphomandra betacea) Epicarp Extracts Obtained by Sustainable Extraction Process
Source: Front Nutr. 2021 Nov 15;8:769617. doi: 10.3389/fnut.2021.769617 (PMC8634709; doi:10.3389/fnut.2021.769617)
Supplement: Supplementary file 1 [file Data_Sheet_1.pdf]

Supplementary Material

Table S1 ANOVA for response surface modeling showing linear, quadratic and interaction relations of each response variable and coefficients of regression equation for model prediction.

Response variable	Factor	DF	SS	*F*-value	*p*-value	CE	
Extraction yield (%)	Model	3893.869	8	15.420	0.024*	β_0	38.61
	SC (% EtOH)	2343.536	1	48.199	0.006*	β_1	-0.06
	SC × SC	114.005	1	2.345	0.223	$\beta_{1,1}$	0.00
	T (°C)	1275.750	1	26.238	0.014*	β_2	0.09
	T × T	11.956	1	0.246	0.654	$\beta_{2,2}$	6.79×10^{-4}
	SC × T	2.756	1	0.057	0.827	$\beta_{1,2}$	-2.77×10^{-4}
	Error	145.866	3				
	$R^2 = 0.963$						
TPC (mg GAE/g)	Model	4934.783	8	5.500	0.096	β_0	22.20
	SC (% EtOH)	1928.803	1	11.924	0.041*	β_1	-0.23
	SC × SC	252.803	1	1.563	0.300	$\beta_{1,1}$	4.50×10^{-3}
	T (°C)	2145.048	1	13.261	0.036*	β_2	-0.15
	T × T	73.706	1	0.456	0.548	$\beta_{2,2}$	1.69×10^{-3}
	SC × T	49.151	1	0.304	0.620	$\beta_{1,2}$	1.17×10^{-3}
	Error	485.271	3				
	$R^2 = 0.902$						
TFC (mg Q/g)	Model	18.505	8	20.530	0.016*	β_0	1.29
	SC (% EtOH)	6.592	1	37.635	0.009*	β_1	-1.63×10^{-2}
	SC × SC	3.175	1	18.129	0.024*	$\beta_{1,1}$	5.04×10^{-4}
	T (°C)	6.448	1	36.811	0.009*	β_2	-3.17×10^{-2}
	T × T	1.334	1	7.618	0.070	$\beta_{2,2}$	2.27×10^{-4}
	SC × T	0.431	1	2.458	0.215	$\beta_{1,2}$	-1.09×10^{-4}
	Error	0.525	3				
	$R^2 = 0.972$						
TCC (mg CE/g)	Model	11348.465	8	18.810	0.018*	β_0	77.99
	SC (% EtOH)	6323.806	1	54.080	0.005*	β_1	-0.56
	SC × SC	2386.044	1	20.405	0.020*	$\beta_{1,1}$	1.38E-02
	T (°C)	770.379	1	6.588	0.083	β_2	-1.53
	T × T	1446.017	1	12.366	0.039*	$\beta_{2,2}$	7.47E-03
	SC × T	71.413	1	0.611	0.492	$\beta_{1,2}$	-1.41E-03
	Error	350.806	3				
	$R^2 = 0.969$						
ABTS (IC_{50} mg/mL)	Model	151.932	8	27.610	0.010*	β_0	8.17
	SC (% EtOH)	4.084	1	3.791	0.147	β_1	9.10×10^{-3}
	SC × SC	2.622	1	2.434	0.217	$\beta_{1,1}$	-4.58×10^{-4}
	T (°C)	107.273	1	99.590	0.002*	β_2	0.19
	T × T	33.702	1	31.288	0.011*	$\beta_{2,2}$	-1.14×10^{-3}
	SC × T	1.020	1	0.947	0.402	$\beta_{1,2}$	1.68×10^{-4}
	Error	3.231	3				
	$R^2 = 0.979$						
LOX (IC_{50} mg/mL)	Model	22088.054	8	2.890	0.206	β_0	40.23
	SC (% EtOH)	4651.507	1	3.672	0.151	β_1	2.48
	SC × SC	1569.867	1	1.239	0.347	$\beta_{1,1}$	-1.12×10^{-2}
	T (°C)	9748.570	1	7.695	0.069	β_2	0.92
	T × T	713.790	1	0.563	0.507	$\beta_{2,2}$	-5.25×10^{-3}
	SC × T	1603.602	1	1.266	0.342	$\beta_{1,2}$	-6.67×10^{-3}
	Error	3800.717	3				
	$R^2 = 0.828$						
ACHE (IC_{50} mg/mL)	Model	41607.445	8	20.650	0.016*	β_0	203.83
	SC (% EtOH)	4274.824	1	10.917	0.046*	β_1	-0.73
	SC × SC	37.109	1	0.095	0.778	$\beta_{1,1}$	-1.72×10^{-3}

T (°C)	28917.528	1	73.848	0.003*	β_2	2.59
T × T	6862.133	1	17.524	0.025*	$\beta_{2,2}$	-1.63×10^{-2}
SC × T	341.110	1	0.871	0.419	$\beta_{1,2}$	3.08×10^{-3}
Error	1174.742	3				
$R^2 = 0.972$						

DF: Degree of freedom; CE: Coefficients of regression equation (Uncoded units); SS: Sum of squares; *Significant.

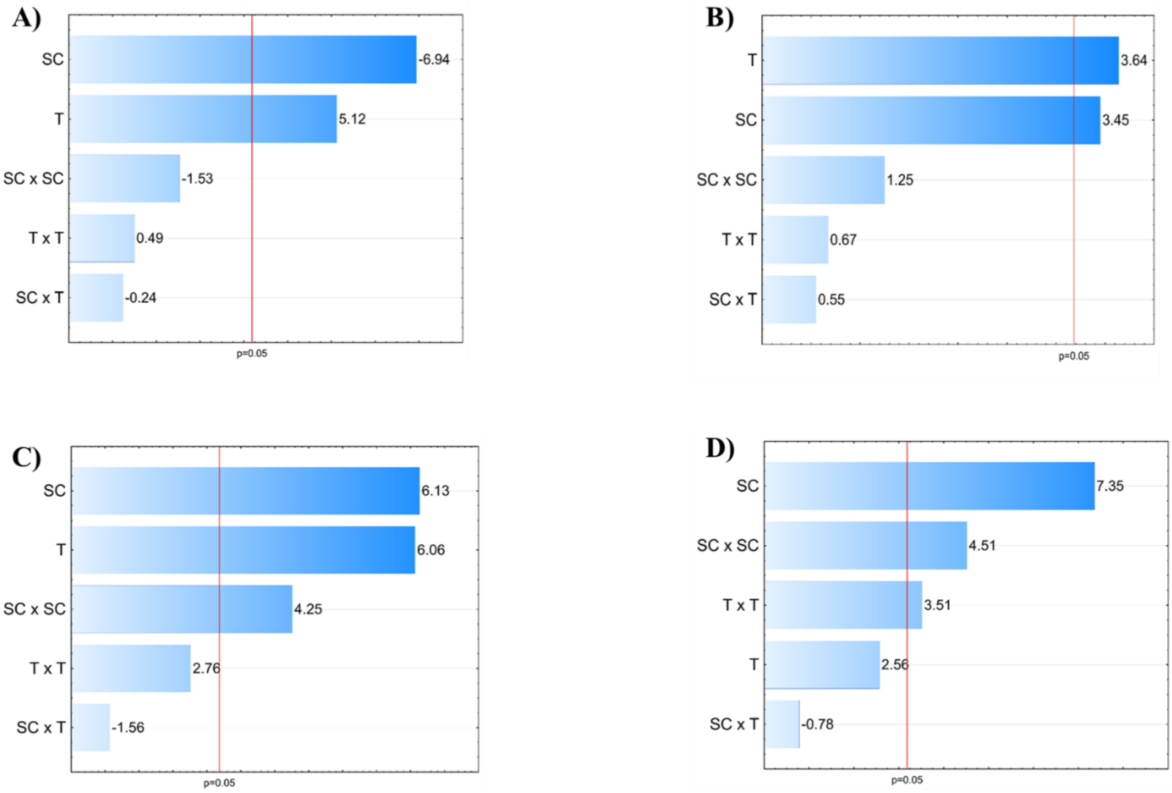

Figure S1 Pareto diagram for effect of solvent composition and temperature in A) total yield, B) TPC, C) TFC and D) TCC of tamarillo epicarp obtained by PLE.

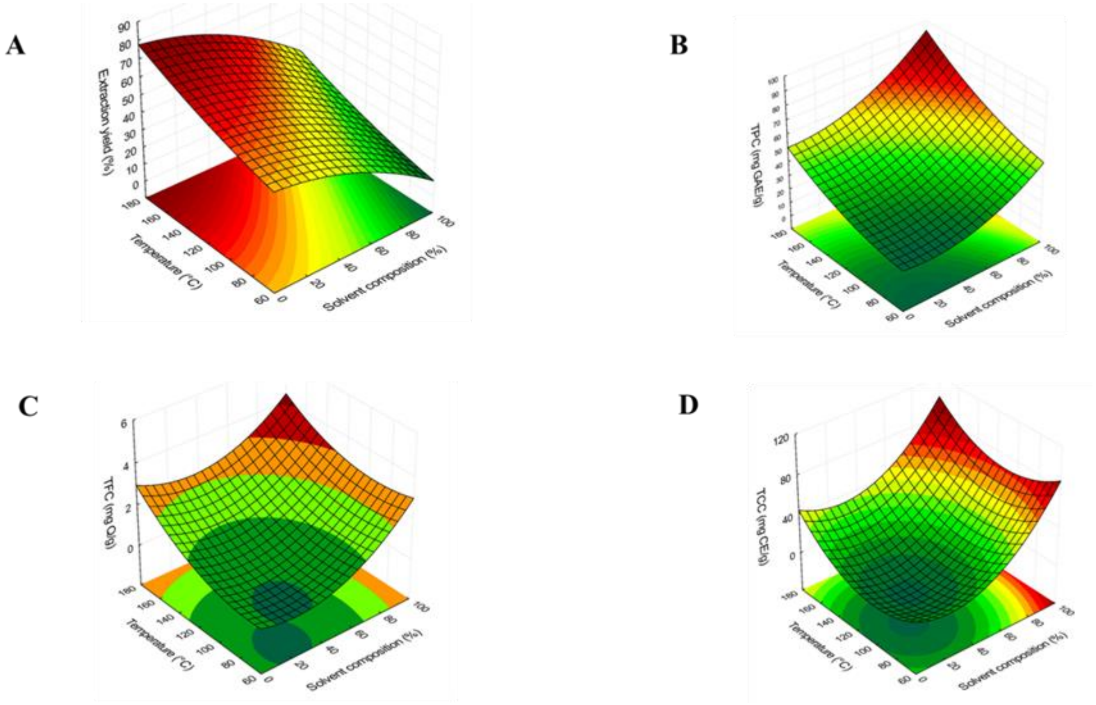

Figure S2 Response surface plot for **(A)** Extraction Yield (%); **(B)** TPC; **(C)** TFC and **(D)** TCC.

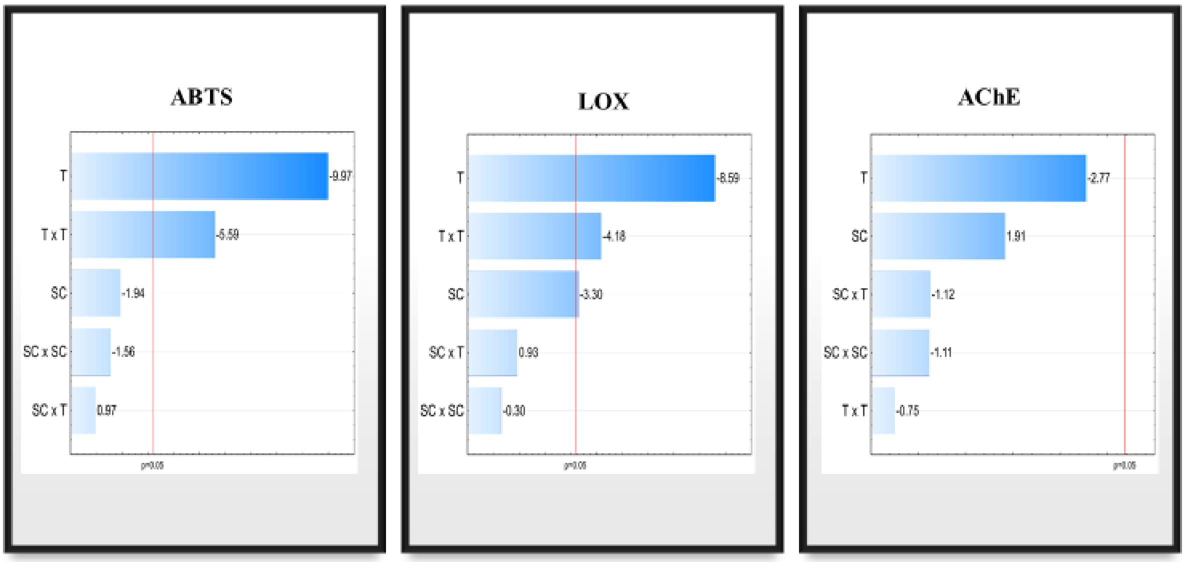

Figure S3 Pareto diagram for effect of solvent composition and temperature in antioxidant activity by ABTS, anti-inflamatory activity by LOX and acetylcholinesterase inhibition enzyme AChE of tamarillo epicarp obtained by PLE.

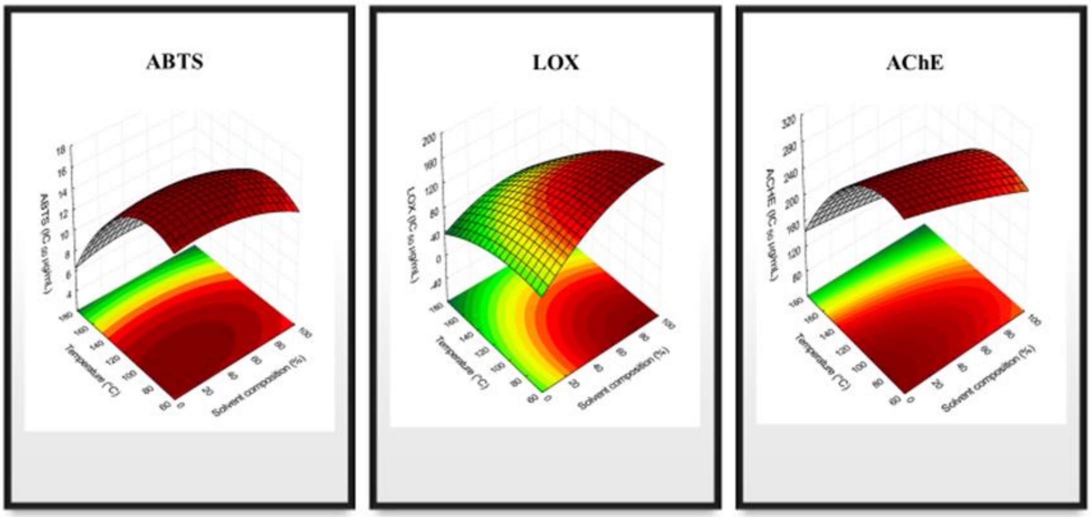

Figure S4 Response surface for biological activities of tamarillo epicarp extracts (ABTS, LOX and AChE) as a function of temperature and solvent composition.
